# Clinical Implications of the Genetic Architecture of Dilated Cardiomyopathy

**DOI:** 10.1007/s11886-020-01423-w

**Published:** 2020-10-10

**Authors:** Lisa D. Wilsbacher

**Affiliations:** 1 Feinberg Cardiovascular and Renal Research Institute, Northwestern University Feinberg School of Medicine, Simpson Querrey Biomedical Research Center 8-404, 303 E. Superior St, Chicago, IL 60611 USA; 2 Department of Medicine, Northwestern University Feinberg School of Medicine, Chicago, IL USA; 3 Department of Pharmacology, Northwestern University Feinberg School of Medicine, Chicago, IL 60611 USA

**Keywords:** Dilated cardiomyopathy, Genetic, Genetic testing, Heart failure, Mutation, Variant

## Abstract

**Purpose of Review:**

Dilated cardiomyopathy (DCM) frequently involves an underlying genetic etiology, but the clinical approach for genetic diagnosis and application of results in clinical practice can be complex.

**Recent Findings:**

International sequence databases described the landscape of genetic variability across populations, which informed guidelines for the interpretation of DCM gene variants. New evidence indicates that loss-of-function mutations in filamin C (*FLNC*) contribute to DCM and portend high risk of ventricular arrhythmia.

**Summary:**

A clinical framework aids in referring patients for DCM genetic testing and applying results to patient care. Results of genetic testing can change medical management, particularly in a subset of genes that increase risk for life-threatening ventricular arrhythmias, and can influence decisions for defibrillator therapy. Clinical screening and cascade genetic testing of family members should be diligently pursued to identify those at risk of developing DCM.

## Introduction

**Fig. 1:**
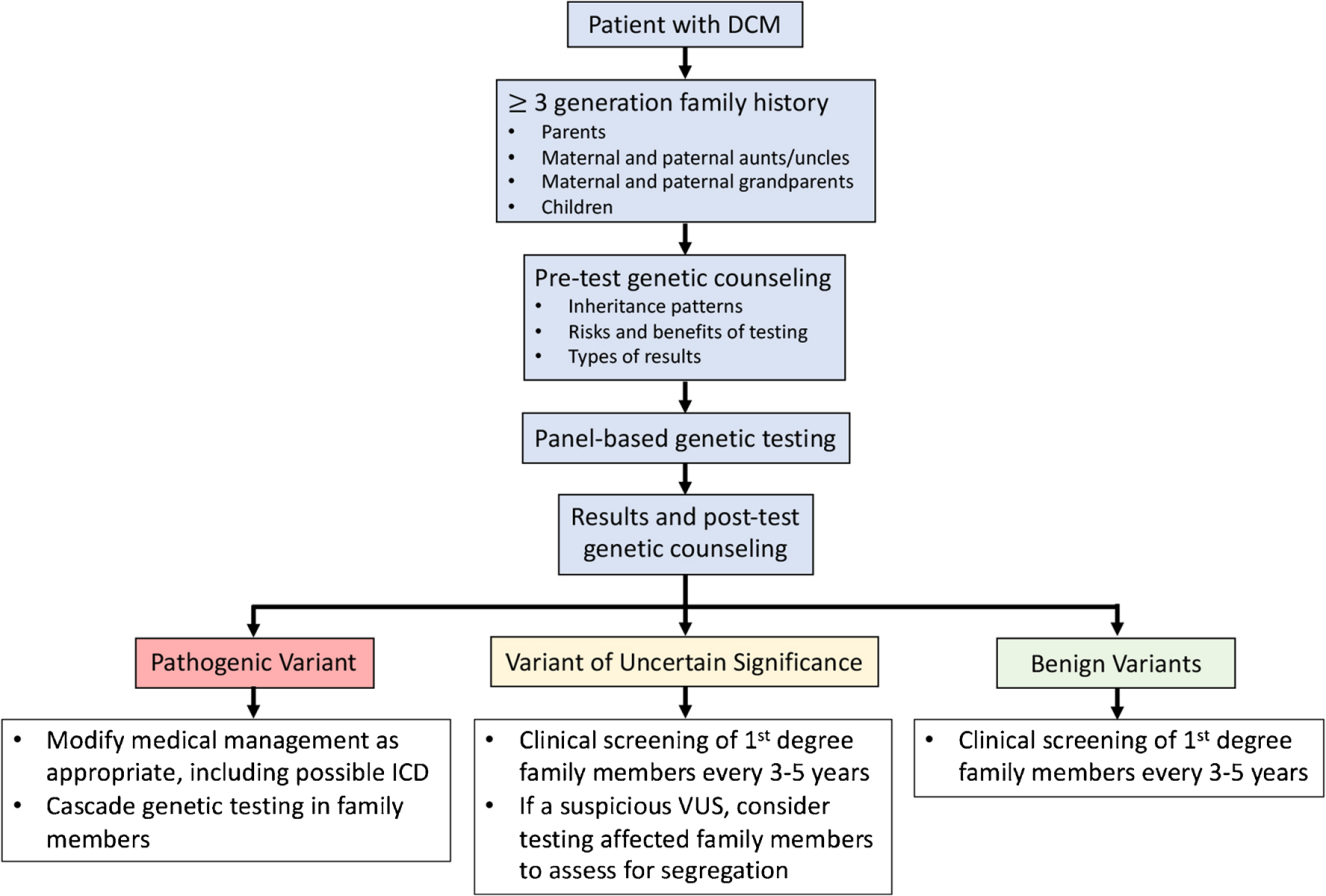
Framework for genetic testing in dilated cardiomyopathy (DCM). ICD, implantable cardioverter defibrillator; VUS, variant of uncertain significance

Dilated cardiomyopathy (DCM) is characterized by an enlarged ventricular cavity with reduced contractile function. By this morphological definition, DCM is often divided into “ischemic” and “nonischemic”; however, the American Heart Association and European Society of Cardiology more strictly define DCM as cardiac enlargement and dysfunction in the absence of hypertension, valvular disease, and ischemia [1, 2]. Approximately 30% of DCM was classified as idiopathic in a large survey of hospitalized patients in the USA, and a genetic cause is suspected in many cases of idiopathic and familial DCM [3]. However, the precise genetic etiology is determined in only ~ 15–35% of individuals with DCM, which illustrates the genetic heterogeneity of DCM [4, 5]. The decision to pursue genetic testing, the interpretation of results, and the appropriate clinical actions based on those results are complex. This review will address these complexities and provide a framework for applying DCM genetic testing in clinical practice (Fig. 1).

## Genetics 101

The human genome contains 3 billion base pairs in each cell of the body, and about 3 million base pairs are different from one unrelated person to another [6]. DNA variants occur throughout the genome. However, a small percentage of variants, generally those in gene coding regions, represent the majority of variants that contribute to DCM. Single nucleotide variants that cause disease are often very rare in the population and have a large effect on phenotype. Loss-of-function (LOF) variants include the following: (1) insertions and deletions that result in a frameshift and downstream premature stop codon, (2) nucleotide substitutions that change an amino acid to a premature stop codon, and (3) canonical splice site variants that alter mRNA splicing. Missense variants lead to a non-terminating amino acid substitution within the encoded protein, with subsequent effects that range from minimal to large. Overall, missense variants that cause DCM have deleterious effects on protein structure and function. While LOF variants result in a phenotype for many genes, these results require careful assessment; for example, missense variants in *MYH7* contribute to DCM, while LOF variants in *MYH7* have not been observed as major drivers of DCM [7].

Chromosome pairs contain genomic DNA; in humans, 22 pairs comprise the autosomal chromosomes, while one chromosome pair (X and Y) refers to the sex chromosomes. In addition, mitochondria contain a distinct genome that encodes a subset of proteins involved in oxidative phosphorylation. Autosomal dominant and autosomal recessive inheritance patterns pertain to genes on autosomal chromosomes, while X-linked dominant and X-linked recessive inheritances refer to genes on the X chromosome. Because mitochondria are maternally inherited from the oocyte, mitochondrial inheritance occurs through the mother.

A person’s DNA sequence (genotype) and their observable traits (phenotype) are related. However, the presence of the same gene mutation may result in different phenotypes among individuals. Penetrance describes the likelihood of a particular mutation to cause disease; if not all individuals with the mutation show a disease phenotype, then the gene has reduced penetrance. Variable expressivity describes the breadth of phenotypes that are found with the same gene mutation. Several factors affect penetrance and expressivity of a particular gene mutation: secondary variants within the genome, epigenetic modulation, and environmental exposures all contribute to the phenotypic heterogeneity of DCM [8].

## Brief Overview of DCM

DCM is defined as an enlarged left ventricle with reduced contractile function in the absence of hypertension, valvular disease, or ischemia [1, 2]. DCM prevalence was reported as ~ 1:2500 in an epidemiological study from 1974 to 1985 in Olmsted County, MN, USA, but more recent analyses estimate a prevalence of ~ 1:250 [8, 9]. By definition, the diagnosis of DCM requires imaging to reveal a dilated ventricle and ejection fraction of < 50%. Additional testing evaluates for ischemia, hypertension, valvular abnormalities, metabolic disease, endocrine disease, and exposure to toxins; idiopathic DCM refers to the absence of these etiologies [1]. Primary management of DCM includes beta blockers, angiotensin-converting enzyme (ACE) inhibitors, angiotensin II receptor blockers (ARBs) without or with neprolysin inhibition (ARNI), and sodium-glucose cotransporter 2 (SGLT2) inhibitors [10, 11]. Implantable cardioverter defibrillator (ICD) therapy is indicated for patients with an ejection fraction of ≤ 35% and New York Heart Association Class II–III symptoms despite optimal medical management [12].

While medical therapies have improved outcomes, the mortality rate of DCM remains high at 3.6 per 100 patient-years in men and 2.3 per 100 patient-years in women [13].

## Approach for DCM Genetic Testing

### Why Test?

Many factors drive the decision for genetic testing in DCM despite the current lack of gene therapies. First, genetic testing may provide a diagnosis. Second, the results of genetic testing can change medical management. For example, patients with mutations in *LMNA* carry a high burden of ventricular tachycardia, and ICD therapy is recommended by the European Society of Cardiology guidelines [14, 15]. Third, cascade genetic testing in family members can identify those at risk of developing cardiomyopathy. Fourth, results of genetic testing can be used for prenatal genetic counseling and preimplantation genetic diagnosis. Finally, genetic testing is cost-effective: A human whole genome can be sequenced for approximately $1000 in 2019 [16].

### Who to Test?

Patients diagnosed with DCM at a young age, especially those with a family history of DCM, are at increased risk of a genetic etiology. Pathogenic (i.e., disease-causing) gene variants have been observed in 15–25% of individuals with isolated idiopathic DCM and in 20–40% of individuals with familial DCM [4, 17•, 18]. Therefore, any individual with DCM diagnosed by the age of 60 should be referred for genetic testing. Patients with a family history of DCM in a first-degree relative should undergo clinical screening with echocardiography; a family member with any ventricular abnormality should also be referred for genetic testing [19]. In keeping with this concept, isolated left ventricular dysfunction (defined as reduced ejection fraction without left ventricular dilation) has been associated with an increased burden of pathogenic variants in DCM-causing genes [20], and it is reasonable to refer individuals younger than 40 with unexplained left ventricular dysfunction.

Persistent tachycardia, alcohol, or chemotherapeutic agents are well-described causes of DCM, yet not all patients exposed to these cardiac stressors develop DCM; in some individuals, an underlying genetic variant may lower the threshold for DCM [1]. Likewise, genetic etiologies were found to contribute to a portion of peripartum cardiomyopathy (PPCM) without recovery of systolic function [21–23]. Because the presence of a DCM pathogenic variant has a large impact on management for the patient and family members, the threshold for genetic testing should be low in the clinical scenarios described above. On the other hand, the role for genetic testing remains unclear for patients with transient DCM in the setting of critical illness or isolated PPMC with recovered systolic function; in these cases, referral for genetic counseling and shared decision-making regarding genetic testing is a reasonable approach.

### How are genetic testing results interpreted, and what types of results are possible?

Genetic testing does not yield a simple “positive” or “negative” result; rather, results must be interpreted in the context of population, segregation, computational, and functional data. Public databases of human genetic variation, including the Genome Aggregation Database (gnomAD; http://gnomad.broadinstutite.org) and ClinVar (https://www.ncbi.nlm.nih.gov/clinvar), provide population data. The Genome Aggregation Database comprises over 125,000 exome sequences and over 15,000 whole genome sequences from populations around the world [24••]. ClinVar provides interpretations and supporting evidence for variants identified during genetic testing [25]. The MalaCards database (https://www.malacards.org) integrates data from 74 sources to generate rich clinical and genetic annotation for human diseases [26]. The Clinical Genome Resource (ClinGen; https://www.clinicalgenome.org/) presents expertly curated reports of clinically relevant genes and their variants; *MYH7* represents the first cardiomyopathy gene with a variant analysis in ClinGen [27, 28]. In terms of segregation data, three-generation family histories provide correlations between genotype and phenotype. The American College of Medical Genetics and Genomics and the Association for Molecular Pathology (ACMG/AMP) created an assessment scheme based on these data, which is used to classify a genetic variant as benign, likely benign, variant of uncertain significance, likely pathogenic, and pathogenic [29••].

While the details of the ACMG/AMP variant classification scheme are beyond the scope of this review, several key features can help clinicians understand the clinical implications of genetic testing results. A “negative” result on gene panel testing means that only Benign variants were observed for all the genes on that panel. Importantly, a negative genetic test does not rule out a genetic cause; the patient may have a DCM-causing mutation in a gene not yet on the panel, and family members remain at risk for genetic DCM. A pathogenic variant (mutation) is one with strong evidence that the variant causes disease. Examples of strong evidence include segregation of the genotype with phenotype in at least 5 family members across three generations, multiple independent observations of the variant associated with the phenotype in the literature, and experimental data that demonstrate loss of protein function or abnormal protein function in the presence of the variant [29••].

A variant of uncertain significance (VUS) represents a nucleotide change in a gene that is observed at a very low population frequency (less than 1 in 100,000 individuals), but the specific nucleotide change is not known to contribute to the disease phenotype. Importantly, a VUS is not the same as a benign result and should not automatically be considered absent of risk. The interpretations provided by genetic testing laboratories are performed with minimal clinical information about the patient; by contrast, the clinician is able to apply detailed knowledge of the patient’s age at diagnosis, family history, and clinical course to refine their interpretation of a VUS. A VUS might be considered “suspicious” or “trending pathogenic” if the variant is not observed in the Genome Aggregation Database and multiple family members have DCM. If the VUS segregates with a DCM phenotype in enough family members, then that VUS can be reclassified as a likely pathogenic variant [30].

### Importance of Genetic Counseling

Genetic counseling should be a part of all genetic testing. Counseling involves a discussion of (1) inheritance patterns; (2) the types of results; (3) potential benefits including genetic diagnosis, changes in medical management, and family testing; and (4) risks of testing. In terms of risks, the 2008 Genetic Information Nondiscrimination Act (GINA) prohibits employers and health insurance companies from using a person’s genetic information to discriminate; however, life insurance companies are not prohibited from seeking a person’s genetic testing results to alter rates [31]. Counseling on these topics should be completed before testing is sent, and additional counseling occurs when results are returned to the patient. Specifically, if a pathogenic or likely pathogenic variant is detected, then testing for that variant should be offered to all first-degree family members. The lack of a pathogenic or likely pathogenic variant does not rule out a genetic cause, as variants in genes not known to cause DCM may be present; in this situation, the patients should inform their first-degree family members, and those family members should undergo clinical screening for DCM every three to five years. Genetic counselors represent a critical resource for providing this information to patients [32].

Genetic testing results may drive additional post-test counseling and recommendations from the cardiologist, such as consideration for ICD therapy and exercise restriction. For individuals with DCM and a pathogenic/likely pathogenic variant in a gene correlated with life-threatening arrhythmias (*LMNA, FLNC, RBM20, DSP*), exercise should be restricted to recreational activities [33]. By contrast, patients with DCM and a pathogenic/likely pathogenic variant in other genes may compete in sports if they are asymptomatic, ejection fraction ≥ 40%, late gadolinium enhancement ≤ 20% on MRI, and no history of unexplained syncope or significant ventricular ectopy on ambulatory ECG and exercise stress testing [33, 34]. Finally, family members identified as genotype-positive but phenotype-negative may participate in competitive sports, but they should undergo annual clinical screening [33].

## Genes Implicated in Monogenic Dominant DCM

The likelihood of detecting a likely pathogenic or pathogenic variant using a cardiomyopathy gene panel had been reported at ~ 37% for DCM, although this analysis was completed before publication of large international sequence databases [4, 24••, 35]. More recently, the Dilated Cardiomyopathy Precision Medicine Study reported a likely pathogenic or pathogenic variant in 15 of 97 (15.5%) probands [5]. This relatively low testing yield reflects the complexity of the genetic architecture of DCM, and it indicates that novel genes and variants underlying DCM remain to be discovered.

A number of genes with definitive and putative contributions to DCM have been described, and these genes encode proteins that function within the sarcomere, Z disc, cytoskeleton, desmosomes, organelles, and extracellular matrix (Table 1). Genes initially attributed to other types of cardiomyopathy, such as *MYBPC3* for hypertrophic cardiomyopathy (HCM) and *DSP* for arrhythmogenic right ventricular cardiomyopathy (ARVC), have been shown to contribute to DCM as well [19, 36, 37•].

**Table 1:** Genes implicated in DCM

Gene	Protein
Sarcomere	
*MYH6†*	α-Myosin heavy chain
MYH7*†	β-Myosin heavy chain
TPM1*†	α-Tropomyosin
ACTC1*†	α-Cardiac actin
TNNT2*†	Cardiac troponin T
TNNC1*	Cardiac troponin C
*TNNI3†*	Cardiac troponin I
*MYBPC3†*	Myosin-binding protein C
TTN*†	Titin
Z disk	
*ACTN2†*	α-Actinin 2
BAG3*†	BCL2-associated athanogene 3
*CRYAB*	α-B-crystallin
*TCAP†*	Titin-cap/telethonin
*MYPN*	Myopalladin
*CSRP3†*	Muscle LIM protein
NEXN*†	Nexilin
*ANKRD1†*	Cardiac ankyrin repeat protein
LDB3*†	Cypher/ZASP
*NEBL*	Nebulette
Cytoskeleton	
DES*†	Desmin
VCL*†	Metavinculin
*FLNC*	Filamin C
Desmosomes	
DSC2	Desmocollin 2
*DSG2†*	Desmoglein 2
DSP*†	Desmoplakin
Dystrophin complex	
DMD*†	Dystrophin
*DTNA*	α-Dystrobrevin
*SGCD†*	δ-Sarcoglycan
*ILK*	Integrin-linked kinase
*FKTN†*	Fukutin
Ion channels	
SCN5A*†	Type V voltage-gated cardiac Na channel
*ABCC9†*	Sulfonylurea receptor 2A, component of ATP-sensitive potassium channel
*HCN4*	Hyperpolarization-activated cyclic nucleotide-gated potassium channel 4
Sarcoplasmic reticulum and cytoplasm	
PLN*†	Phospholamban
*RYR2*	Ryanodine receptor 2, Ca channel
*DOLK*	Dolichol kinase
*RAF1†*	Proto-oncogene
Nuclear envelope	
LMNA*†	Lamin A/C
*EMD*	Emerin
*TMPO†*	Lamin-associated polypeptide 2
*SYNE1/2*	Nesprin 1/2
Nucleus	
*EYA4†*	Eyes absent 4
*PRDM16*	PR domain containing 16
*TBX20*	T-box 20
RBM20*†	RNA-binding protein 20
*GATAD1*	GATA zinc finger domain protein 1
*NKX2-5†*	Cardiac-specific homeobox 1
*LRRC10*	Leucine-rich repeat containing 10
Mitochondria	
*TAZ/G4.5†*	Tafazzin
*TXNRD2*	Thioredoxin reductase 2
Lysosome	
*LAMP2†*	Lysosome-associated membrane protein 2
Extracellular matrix	
*LAMA4*	Laminin 4
Other	
*CHRM2*	Cholinergic receptor muscarinic 2
*MIB1*	Mitogen-activated protein kinase kinase 2
*TTR*	Transthyretin

List represents 55 genes that are offered on multiple commercial DCM genetic testing panels. Asterisks indicate genes with very strong evidence of pathogenicity as determined by multiple independent groups. [5, 38–41]. Daggers indicates a MalaCards score greater than 100 [26]

As the lists of genes in commercial DCM panels grow, expert curation adds context for interpretation. Independent groups have identified genes that consistently accounted for pathogenic and likely pathogenic variants for DCM: *TTN, MYH7, DSP, SCN5A, LMNA, TPM1, TNNC1, TNNT2, BAG3, PLN, RBM20, LDB3, DMD, DES, ACTC1, NEXN,* and *VCL* [5, 38–41]. *FLNC* was subsequently identified as an important gene for DCM and should be included in this group [5]. In these analyses, a novel or rare variant in large population sequence databases (i.e., gnomAD) provided the first level of analysis.

Ranking genes for evidence of pathogenicity significantly increased the odds ratio of a variant appearing in an individual with DCM; the highest ranked group of genes required evidence of segregation in 5 or more family members, in vitro functional studies, and heterozygous or humanized variant animal models [40]. Each of these criteria are components of the ACMG/AMP variant classification scheme, but they are not strictly required together for a variant to meet likely pathogenic or pathogenic classification [29••]. The Heart Failure Society of America and the American Heart Association each published clinical practice resources for DCM genetic testing, which recommended testing for *TTN*, *LMNA*, *MYH7*, *TNNT2*, *BAG3*, *RBM20*, *TNNC1*, *TNNI3*, *TPM1*, *SCN5A*, and *PLN* [17•, 18, 42•]. However, next-generation sequencing technology allows for the sequencing of dozens of genes in parallel at virtually no increased cost. Therefore, when choosing a genetic testing panel, using the largest available panel will maximize the likelihood of detecting a likely pathogenic or pathogenic variant. This approach also increases the likelihood of variants of uncertain significance, but observational studies do not detect an adverse effect on patients upon learning of VUSs [43, 44]. Furthermore, the detection of suspicious VUSs creates an opportunity for resolution of the VUS toward pathogenic or benign if other affected family members are tested. In keeping with this concept, the Heart Failure Society of America resource recommended the inclusion of HCM and ARVC genes when testing for DCM, with the acknowledgment that a larger number of VUSs will be identified [45].

## Genes Implicated in DCM: Recent Updates

### Titin

*TTN* encodes the largest protein (~ 35,000 amino acids) expressed in the body. TTN protein spans one-half of the sarcomere from the Z disc to the M line and comprises four domains: the Z disc binding region, I band domain that overlaps sarcomeric actin filaments, A band domain that overlaps myosin filaments, and the M line binding region. In 2012, the *TTN* gene sequence was published, and truncating variants in the A band region of *TTN* (*TTN*tv) were found to account for ~ 15–20% of all DCM [46••, 47]. At the same time, up to 3% of apparently healthy controls harbor *TTN*tvs, which demonstrates variable penetrance of these variants [48]. Furthermore, a recent single-center study found enrichment of TTNtvs in individuals of European ancestry with DCM, but unexpectedly these *TTN* truncating variants were not enriched in individuals of African ancestry in their DCM cohort [47]. Despite these complexities, *TTN*tvs carry prognostic significance: multiple recent studies associated *TTN*tvs with recovery of left ventricular systolic function and improved outcomes in the setting of guideline-directed medical therapy [49–52].

### RNA-Binding Protein Motif 20 (RBM20)

*RBM20* is an RNA splicing factor enriched in cardiomyocytes and skeletal muscle that regulates splicing of *TTN*, calcium/calmodulin dependent protein kinase II delta (*CAMK2D*), and ryanodine receptor 2 (*RYR2*); individuals with pathogenic variants in *RBM20* are at high risk of DCM and ventricular arrhythmias [53–56]. Further investigation of patients from an international registry identified two regions in exons 9 and 11 in *RBM20* with significant enrichment for variants associated with cardiomyopathy, ventricular and atrial arrhythmias, and sudden cardiac death [57•]. An independent study of 15 Dutch families with *RBM20* pathogenic variants, all within the exon 9 and exon 11 enriched regions, found 66% penetrance of DCM and 30% with significant ventricular arrhythmia or sudden death [58]. The arrhythmogenic nature of *RBM20* pathogenic variants, particularly those in exons 9 and 11, should prompt a discussion of early ICD implantation.

### Desmoplakin

*DSP* encodes desmoplakin, a component of the desmosome that is highly expressed in the skin and cardiomyocytes. Pathogenic variants in *DSP* were originally described in patients with autosomal dominant ARVC, but subsequent small case series reported both missense variants and truncating variants associated with left-dominant arrhythmogenic DCM [59–65]. A large case series of 107 individuals with *DSP* pathogenic variants (105 with truncating variants) revealed left ventricular phenotypes in 86%, including dilation, delayed enhancement, and myocardial injury; while ventricular arrhythmias were found in 56% of individuals with *DSP* mutations, diagnostic criteria for ARVC were met less frequently for *DSP* positive as compared with *PKP2*-positive individuals [37•]. Myocardial injury and a distinct pattern of diffuse subepicardial delayed enhancement, even before the onset of left ventricular dilation, appear to be specific clinical findings for *DSP*-mediated cardiomyopathy [37•, 64, 66].

### Filamin C

*FLNC* encodes an actin-binding intermediate filament that is highly expressed in cardiomyocytes and skeletal muscle and links membrane proteins with sarcomeres. Missense variants in *FLNC* have been previously associated with skeletal muscle myofibrillar myopathy and hypertrophic cardiomyopathy, but recent studies identified *FLNC* truncating variants (*FLNC*tvs) as an important driver of arrhythmogenic DCM [67••]. In the initial study of 28 probands with *FLNC*tvs and their genotype-positive family members, Ortiz-Genga et al. found an alarming prevalence of ventricular arrhythmias (82%) and sudden cardiac death (40 cases in 21 of 28 families) [67••]. Functional studies supported *FLNC* haploinsufficiency as the mechanism driving arrhythmogenic DCM [68, 69]. Multiple groups have reported *FLNC*tvs in probands and families with DCM and ventricular arrhythmias [68–74], which provides strong evidence for *FLNC*tv pathogenicity in arrhythmogenic DCM.

These recent developments in the genetic architecture of DCM underscore the importance of genetic evaluation in DCM. *LMNA* and *SCN5A* have long known to be associated with life-threatening ventricular arrhythmias, and the studies of *RBM20, DSP,* and *FLNC* discussed here clearly demonstrate significant arrhythmia and sudden death risks. In terms of clinical management, defibrillator therapy should be considered early in patients with *LMNA, SCN5A, RBM20, DSP,* or *FLNC* pathogenic variants, even before significant dilation and left ventricular systolic dysfunction have occurred.

## Emerging Concepts in DCM Genetic Testing

### Effects of population distributions within sequence databases on variant interpretation

The likelihood of detecting a likely pathogenic or pathogenic variant in DCM is ~ 15–25% for isolated DCM and ~ 20–40% for familial DCM, which reflects the complexity and genetic heterogeneity of DCM [4, 5, 35]. Importantly, genetic variation across populations affects variant interpretation. The vast majority of early genetic testing was performed in individuals of European non-Finnish ancestry; as worldwide testing across diverse populations has increased, many variants observed at low frequency in the European Non-Finnish population were observed in other populations at higher frequency than predicted for a rare disease. Analysis of cardiomyopathy testing at the Laboratory for Molecular Medicine revealed a lower likelihood of detecting a likely pathogenic or pathogenic variant—and a higher likelihood of a VUS—in individuals of African ancestry [75]. Furthermore, in individuals of African and Latino ancestry, VUSs in medically actionable cardiomyopathy genes are associated with clinical findings such as increased left ventricular diameter in systole and diastole [76]. As described above in a single DCM referral center, *TTN* truncating variants were enriched in individuals of European ancestry but not in individuals of African ancestry; reasons for this unexpected observation require further study [47]. In 2020, the population distribution in Genome Aggregation Database remained uneven: Individuals of European Non-Finnish ancestry accounted for 45% of exomes and genomes, while individuals of African, Latino, East Asian, and South Asian ancestry accounted for 9%, 13%, 7%, and 11%, respectively (the remaining data comprise Ashkenazi Jewish, Finnish, and “other” ancestries). Accurate interpretation of variants depends upon representative sampling of a sufficient number of individuals across multiple populations; inclusion of more non-European individuals and families remains an ongoing goal within the field [5]. Expansion of genetic testing in non-European individuals and families will improve classification, since many variants meet likely pathogenic or pathogenic classification by combining evidence from multiple families.

### Polygenic Risk Scores

Given the genetic heterogeneity of DCM, it is reasonable to predict that common variants with minor allele frequency of > 1% will partially contribute to DCM phenotypes. Genome-wide association studies (GWAS) have identified several common single nucleotide variants associated with idiopathic DCM at the population level [77–79].

Polygenic risk scores (PRSs) calculate a relative risk of developing disease based on an individual’s number of common variants associated with that disease [80]. Currently, the relatively small number of common variants associated with DCM limits the applications of PRSs, but this application will likely augment DCM risk prediction as additional GWAS are completed.

### Population Databases and Refining Estimates of Penetrance

As population sequence databases have increased in size, efforts have been made to use this information to refine penetrance estimates. This concept holds particular relevance in DCM, where incomplete and age-dependent penetrance remain important concepts in counseling patients who are genotype-positive but phenotype-negative. To date, methods to use population-based whole exome and whole genome sequencing for penetrance estimates show variability [81–83]. The Genome Aggregation Database does not contain phenotypic data, but other programs including NHLBI Trans-Omics for Precision Medicine (TOPMed), NIH *All of Us*, NHGRI Genome Sequencing Project, and Harvard Medical School Genomes2People have been designed to include whole exome or whole genome sequencing with deep phenotyping information. The generation of large datasets linking genotype and phenotype from diverse populations should significantly improve estimates of penetrance.

### Whole-Exome Sequencing and Whole-Genome Sequencing as Initial Genetic Testing Strategies

The most common genetic testing approach utilizes panel-based sequencing. Many genetic testing laboratories offer WES as an alternative approach [84]. However, whole-exome sequencing (WES) may not provide sufficient coverage for all genes; TNNI3 and PLN were reported to have insufficient coverage for complete variant interpretation [85]. By contrast, results from whole-genome sequencing (WGS) correlated well with panel-based sequencing, and WGS performed better than WES: WES covered only 69% of panel sequence targets, most likely due to capture bias during sample preparation and use of predefined target regions that may miss isoforms [86]. Because WES and WGS are not limited to known DCM genes, the provider must be prepared for clinically actionable likely pathogenic and pathogenic variants in non-cardiomyopathy genes. Overall, the practical applications of WES and WGS are still being developed for clinical DCM genetic testing. In the research setting, WGS remains a powerful technique for new DCM gene discovery.

## Conclusion

DCM is a genetically heterogeneous condition, and the interpretation of results can be complex. Despite these complexities, results from genetic testing can have a profound impact on patient management, particularly for the arrhythmogenic subset of DCM. Furthermore, cascade genetic testing can identify family members at risk for developing DCM. Finally, expansion of testing in diverse populations will improve variant interpretation in the future.
